# P-1188. Mortality among U.S. Children Aged < 18 Years Hospitalized with Laboratory-confirmed Respiratory Syncytial Virus (RSV) Infection, 12 States, 2018–2019, 2021–2022, 2022–2023

**DOI:** 10.1093/ofid/ofae631.1372

**Published:** 2025-01-29

**Authors:** Eszter Toth, Michael Whitaker, Jennifer Milucky, Huong Pham, Pam Daily Kirley, LeAnna Kent, Daewi Kim, Kyle P Openo, Alicia Brooks, Lauren Leegwater, Kathryn Como-Sabetti, Chad Smelser, Adam Rowe, Kevin Popham, Melissa Sutton, Keipp Talbot, Ryan Chatelain, Fiona P Havers, Monica E Patton

**Affiliations:** Emory University, Atlanta, Georgia; CDC, Marietta, Georgia; Centers for Disease Control and Prevention, Atlanta, Georgia; Centers for Disease Control and Prevention, Atlanta, Georgia; California Emerging Infections Program, Oakland, California; Colorado Department of Public Health and Environment, Denver, Colorado; Yale CT Emerging Infections Program, New Haven, Connecticut; Georgia Emerging Infections Program and Atlanta VA Medical Center, Decatur, GA; Maryland Department of Health, Baltimore, Maryland; Michigan Department of Health & Human Services, Grand Rapids, Michigan; Minnesota Department of Health, St Paul, MN; New Mexico Department of Health, Santa Fe, New Mexico; New York State Department of Health, Albany, New York; Universirty of Rochester, Rochester, NY; Oregon Health Authority, Portland, Oregon; Vanderbilt University Medical Center, Nashville, Tennessee; Salt Lake County Health Department, Salt Lake City, Utah; CDC, Coronavirus and Other Respiratory Viruses Division, Atlanta, Georgia; CDC, Marietta, Georgia

## Abstract

**Background:**

Pediatric RSV-associated hospitalizations are well-described but mortality data are limited.

**Figure:**
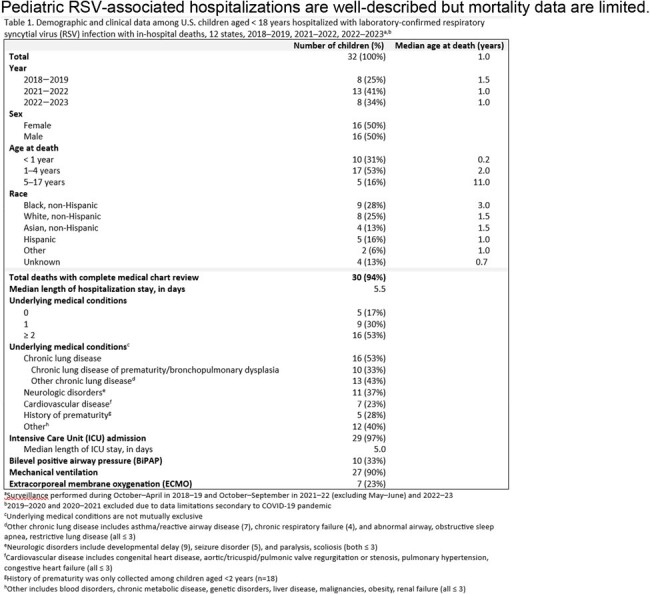

**Methods:**

RSV Hospitalization Surveillance Network (RSV-NET), a 12-state population-based surveillance system, captured laboratory-confirmed RSV hospitalizations among catchment-area residents during October–April in 2018–19 and October–September in 2021–22 (excluding May–June) and 2022–23. Medical charts were reviewed for children who died in-hospital and for those who died ≤30 days post-discharge in 2018–19 via matching case and death certificate data. We described in-hospital and 2018–19 post-discharge mortality among RSV-NET children aged < 18 years. 2019–21 and 2020–21 data were excluded due to COVID-19.

**Figure:**
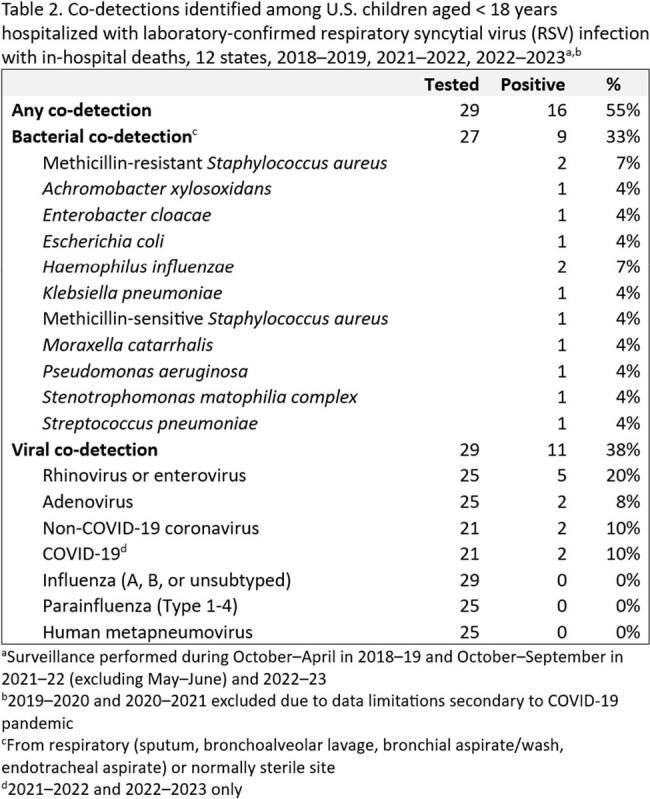

**Results:**

During 2018–19, 2021–22, and 2022–23, 18,511 pediatric RSV hospitalizations were reported with 32 (0.2%) in-hospital deaths among 10, 17, and 5 children aged < 1, 1–4, and 5–17 years, respectively (Table 1). Among 28 children with race and ethnicity data, 9 were non-Hispanic (NH) Black, 8 were NH White, 4 were NH Asian, 5 were Hispanic, and 2 were another race or ethnicity. Almost all (83%) children with medical chart review had ≥ 1 underlying medical condition (UMC); 72% had ≥ 2 UMCs. UMCs included chronic lung disease (CLD) (53%), neurologic disorders (37%), and cardiovascular disease (23%). In all, 33% of children had CLD of prematurity; prematurity, only collected for those < 2 years (n=18 deaths), was noted for 5 (28%) children. Co-detections, including 9 bacterial from respiratory or sterile sites and 11 respiratory viral detections, were noted for 16 (55%) children who died (Table 2).

In 2018–19, 8 in-hospital and 4 post-discharge deaths occurred; the median age at death was 1.5 and 6.5 years, respectively, and the median length of stay was 5.5 and 11.5 days, respectively. All (100%) children had ≥ 1 UMC; ≥ 2 UMCs were noted among 50% and 100% of children with in-hospital and post-discharge deaths, respectively. An RSV-specific cause of death was listed on 4 (33%) of 12 death certificates from 2018–19.

**Conclusion:**

Almost all RSV-NET children who died had ≥ 1 UMC; bacterial or viral co-detections were common. RSV-attributable pediatric deaths are likely underestimated if limited to in-hospital deaths or death certificates with RSV-specific causes.

**Disclosures:**

**All Authors**: No reported disclosures

